# The Population Genetics of dN/dS

**DOI:** 10.1371/journal.pgen.1000304

**Published:** 2008-12-12

**Authors:** Sergey Kryazhimskiy, Joshua B. Plotkin

**Affiliations:** 1 Biology Department, University of Pennsylvania, Philadelphia, Pennsylvania, United States of America; 2 Program in Applied Mathematics and Computational Science, University of Pennsylvania, Philadelphia, Pennsylvania, United States of America; National Institute of Genetics, Japan

## Abstract

Evolutionary pressures on proteins are often quantified by the ratio of substitution rates at non-synonymous and synonymous sites. The dN/dS ratio was originally developed for application to distantly diverged sequences, the differences among which represent substitutions that have fixed along independent lineages. Nevertheless, the dN/dS measure is often applied to sequences sampled from a single population, the differences among which represent segregating polymorphisms. Here, we study the expected dN/dS ratio for samples drawn from a single population under selection, and we find that in this context, dN/dS is relatively insensitive to the selection coefficient. Moreover, the hallmark signature of positive selection over divergent lineages, dN/dS>1, is violated within a population. For population samples, the relationship between selection and dN/dS does not follow a monotonic function, and so it may be impossible to infer selection pressures from dN/dS. These results have significant implications for the interpretation of dN/dS measurements among population-genetic samples.

## Author Summary

Since the time of Darwin, biologists have worked to identify instances of evolutionary adaptation. At the molecular scale, it is understood that adaptation should induce more genetic changes at amino acid altering sites in the genome, compared to amino acid–preserving sites. The ratio of substitution rates at such sites, denoted dN/dS, is therefore commonly used to detect proteins undergoing adaptation. This test was originally developed for application to distantly diverged genetic sequences, the differences among which represent substitutions along independent evolutionary lineages. Nonetheless, the dN/dS statistics are also frequently applied to genetic sequences sampled from a single population, the differences among which represent transient polymorphisms, not substitutions. Here, we show that the behavior of the dN/dS statistic is very different in these two cases. In particular, when applied to sequences from a single population, the dN/dS ratio is relatively insensitive to the strength of natural selection, and the anticipated signature of adaptive evolution, dN/dS>1, is violated. These results have implications for the interpretation of genetic variation sampled from a population. In particular, these results suggest that microbes may experience substantially stronger selective forces than previously thought.

## Introduction

The identification of genetic loci undergoing adaptation is a central project of evolutionary biology. With the advent of sequencing technologies, a variety of statistical tests have been developed to quantify selection pressures acting on protein-coding regions. Among these, the dN/dS ratio is one of the most widely used, owing in part to its simplicity and robustness. This measure quantifies selection pressures by comparing the rate of substitutions at silent sites (dS), which are presumed neutral, to the rate of substitutions at non-silent sites (dN), which possibly experience selection. The ratio dN/dS is expected to exceed unity only if natural selection promotes changes in the protein sequence; whereas a ratio less than unity is expected only if natural selection suppresses protein changes [1],[2]. This intuitive interpretation of dN/dS is supported by theoretical work on the relationship between the dN/dS statistic and the underlying selection pressure in a Wright-Fisher model [3].

The dN/dS ratio was originally developed for the analysis of genetic sequences from divergent species [1],[4],[5], the differences amoung which represent fixation events along independent lineages. Theoretical work on the relationship between dN/dS and selection likewise assumes that sequences are sampled from independent, divergent species [3], as do computer packages used to estimate dN/dS from data [6],[7]. Nonetheless, the dN/dS ratio test is frequently applied to data that may represent samples from a single population, particularly in the case of microbes (e.g. [3], [8]–[18]). In such cases, the differences between sequences do not represent fixation events along independent lineages, but rather polymorphisms segregating in a single population. It is important, therefore, to understand the relationship between selection pressures and the dN/dS statistic for samples from a single population.

Here we analyze the population genetics of dN/dS. We find that the relationship between the selection pressure and dN/dS is qualitatively different for samples drawn from a single population compared to sampled from divergent lineages. As a result, standard tests for selection based on dN/dS are extremely sensitive to violation of the assumption of divergent lineages. We show that the expected dN/dS ratio within a population is relatively insensitive to selection pressure—a result which helps to explain a body of empirical observations about microbial populations. Moreover, we show that the hallmark signature of positive selection across divergent lineages, dN/dS>1, does not hold within population: strong positive selection is expected to produce dN/dS<1 among population samples. As a result, when applied to intra-specific samples, the standard interpretation of dN/dS is unjustified and may lead to surprising conclusions. This point is illustrated by two recent studies that report dN/dS ratios near 1 among strains of *Salmonella enetrica* serovar Typhi [17],[18], and conclude that genetic drift dominates the bacterium's evolution. This conclusion is surprising in light of the large population size of the bacterium (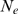 estimated to be on the order of 10^5^) and strong selective advantages of antibiotic-resistance mutations [17]. However, our analysis shows that dN/dS values obtained from closely related isolates may be near 1 under both strong positive selection or moderate negative selection, and so parts of the *Salmonella* Typhi genome may well be evolving under considerable selection pressure.

Our presentation begins with a review of the theory underlying the interpretation of dN/dS across divergent lineages. We then develop the appropriate theory for studying selection and dN/dS within a single population. We compare our theoretical expectations to Monte Carlo simulations based on the Wright-Fisher model. We conclude with a discussion of practical implications.

## Results

### Time-Scales of Adaptation

There are at least two time-scales on which to investigate adaptive evolution: short time-scales, which apply to genetic variation segregating within a population of conspecifics; and long, or evolutionary, time-scales, which apply when comparing the genomes of divergent species.

Over short time-scales, natural selection at a genetic locus may be inferred by inspecting sequences sampled from a population. Polymorphism data are typically compared to expectations under a neutral null model, such as the Wright-Fisher model that forms the basis of Kingman's coalescent [19] and all coalescent-based tests of neutrality [20]–[22]. Alternatively, polymorphism data can be compared to expectations under a Wright-Fisher model that incorporates selection—an approach adopted by the Poisson Random Field method of inferring selection coefficients [23],[24]. Under both of these approaches, the sequences under analysis share a common ancestor within the past 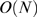 generations, where 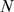 is the population size. Such investigations inform our understanding of the forces that shape genetic variation within a population.

Over long time-scales, by contrast, natural selection is often quantified by comparing orthologous gene sequences from divergent species. In this context, each species is associated with a single representative genetic sequence, and intraspecific polymorphisms are ignored [4]. Instead, the focus is on the rate of substitutions along divergent lineages—i.e. the rate at which mutations arise and subsequently fix. Such investigations inform our understanding of the processes that shape the similarities and differences between the (stereotypical) genomes of divergent species.

Over long time-scales, the dN/dS ratio is an extremely popular measure of adaptive evolution in protein-coding sequences. This measure quantifies selection pressures by comparing the rate of substitutions at silent sites (dS), which are presumed neutral, to the rate of substitutions at non-silent sites (dN), which possibly experience selection. In practice, the dN/dS ratio is commonly estimated from data using, for example, the PAML computer package [7]. Under this approach, the substitution process at a site is described by a continuous-time Markov chain with 61 possible states, corresponding to the 61 sense codons. The instantaneous rate of change from codon *i* to codon *j* depends principally on the parameter *ω*, defined as the relative rate of non-silent versus silent substitutions [2].

The Markov-chain model underlying PAML's calculation of dN/dS explicitly ignores polymorphisms segregating within a population; instead, it represents each divergent species as a single sequence. Furthermore, the Markov-chain model does not describe any details of the process by which a mutation enters a population, changes in frequency, and eventually fixes. Instead, fixation events occur instantaneously in the model, and transient polymorphisms within each divergent population are ignored. These simplifying assumptions are perfectly reasonable when studying substitution rates between long divergent species (e.g. [4]). Over the time-scales of such divergence substitution events are effectively instantaneous.

Given a data set of diverged sequences, and assuming (or simultaneously inferring) their phylogenetic relationship, PAML estimates the parameter *ω* by maximum likelihood. The likelihood function is derived from the Markov chain, assuming that the substitution process at one site is independent of processes at all other sites. It is critical to emphasize that, by definition, *ω* describes the relative rate of selected versus neutral *fixation* events. Therefore, it makes sense to estimate *ω* from a data set of diverged sequences, the differences between which represent fixed substitutions that have accrued along independent branches. But it is not appropriate to estimate *ω* from a set of conspecific sequences sampled from a single population, because the differences between such sequences represent segregating polymorphisms as opposed to fixed substitutions.

### Theory

#### The Relationship between Selection and dN/dS over Long Time-Scales

Although originally formulated without reference to population genetics *per se*, Yang's Markov-chain model of the substitution process at a site can be derived as an appropriate long-time limit of an underlying Wright-Fisher population process [3]. Such a derivation makes two essential assumptions: (1) sites are independent and thus non-interfering; and (2) there are never more than two alleles segregating in a population at a single nucleotide site. The former assumption, of site independence, is shared by most population-genetic models that incorporate selection, such as the Poisson Random Field model. The latter assumption is justified provided that the population-scaled mutation rate is small enough, so that one allelic variant at a site will always fix or go extinct before another allelic variant is introduced. Under these assumptions, the rate of fixation of new mutations with selection coefficient *s* is given simply by the product of the population-scaled mutation rate and the probability of fixation [3]:
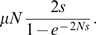
(1)Rates of this form are used as the instantaneous transition rates in the Markov-chain model of substitutions. As a result, if silent substitutions are assumed neutral and all non-silent mutations experience selection coefficient *s*, then the expected ratio of their rates, *ω*, is given by [3]

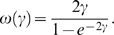
(2)where *γ* is defined as the scaled selection coefficient 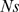.

Equation (2) provides an important link between *ω*, the ratio of substitution rates along independent lineages, and *γ*, the underlying selection coefficient in a Wright-Fisher model. This equation was derived using Kimura's expression for the probability that a new mutation will fix in a population, under a Wright-Fisher model. This derivation is appropriate, because dN/dS is defined as the ratio of fixation rates along independent lineages. We can therefore use Equation (2) in the context of divergent sequences, the differences between which represent fixation events. In particular, Equation (2) provides rigorous meaning to the statement that dN/dS is expected to exceed unity only when there is positive selection to promote non-silent changes: according to Equation (2), *ω* exceeds unity only if *γ* is positive, and *ω* is less than unity only if *γ* is negative.

#### The Population Genetics of dN/dS

Researchers often compute a dN/dS value when comparing conspecific sequences, whose differences reflect polymorphisms segregating within a population (e.g. [3], [8]–[18]). Equation (2) does not apply to such sequences, because differences among such sequences do not represent fixation events along independent lineages. How, then, are we to interpret dN/dS values measured from intraspecific data? What is the relationship between selection and dN/dS values computed for sequences sampled from a population?

To address this question, we must understand the behavior of the dN/dS statistic within a single population over a relatively short time-scale—i.e. the population genetics of dN/dS. In this context, dN and dS represent, respectively, the number of *non-silent mutations* (as opposed to fixations) per non-silent site and the number of *silent mutations* (as opposed to fixations) per silent site, along the coalescent between individuals sampled from the population.

In principle, calculating these quantities requires knowing the expected coalescent time between sampled individuals. Since the general expression for the coalescent time in the presence of selection is not known, we approximate dN and dS by the number of *differences* between two sampled individuals, at non-silent and silent sites respectively. (While the number of mutations along the coalescent between two individuals can be any integer, the number of differences can be only 0 or 1, depending upon whether the two individuals share the same nucleotide at the focal site.) We operate under the same two simplifying assumptions that Nielsen & Yang used in their analysis of dN/dS and selection [3]: (1) sites are assumed independent and non-interacting; and (2) no more than two mutations are assumed to segregate in the population at a single site. The latter approximation will be accurate provided two individuals are typically separated by at most one mutation along their coalescent—i.e. provided that 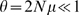. This approximation is justified for most known biological populations, because *θ* per site is typically less than unity.

In order to calculate the expected number of differences between two sampled individuals we utilize the stationary allele frequency distribution at a site. If Φ denotes the stationary frequency distribution for polymorphisms that arise at rate *μ* and experience selection pressure *s*, then we may calculate the expected number of differences per site, denoted *D*:
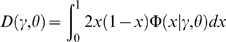
(3)Here *γ* denotes the product 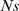, and *θ* denotes 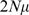.

We use diffusion theory to derive an expression for the stationary frequency distribution of polymorphisms at a site, Φ. In the case of recurrent mutation between two alleles with fixed fitnesses 1 and 1+*s*, the stationary distribution has been solved classically using a zero-flux condition [25],[26]. However, the model of selection analyzed by Yang and other authors (e.g. [3], [4], [27]–[35]) in the context of dN/dS is qualitatively different from the classic model of two alleles under recurrent mutation [25].

Strictly speaking, Yang's model of selection is a special case of an infinite-sites model under which subsequent mutations each provide an additional selective advantage (or disadvantage) *s*. In general, such models are extremely complicated because multiple mutant linages compete with each other [36]–[41]. However, when the mutation rate is small enough, at most two genotypes segregate in the population at any given time, and so the allele frequency dynamics can be described by a simple two-allele Wright-Fisher model. In this limit, the population is monomorphic for the resident allele until a mutant appears. Each mutant has the same selective advantage (or disadvantage) *s* over the resident type. The mutant is either lost or fixed before the next mutant type arises. If the mutant fixes, it becomes the new resident type, and a subsequent mutation will experience the same selective advantage (disadvantage) *s* over the new resident type. This is the model of positive (negative) selection *sensu* Yang [4]. Such a model provides a convenient description of continual positive (or negative) selection at a site, and so we call it the continual selection model.

In the Methods section we derive an expression for the stationary allele frequency distribution under the model of continual selection. The solution is derived by diffusion theory using a constant but non-zero flux condition [42],[43], and it deviates from the classical stationary distribution of Wright [26]. The solution for Φ is given by
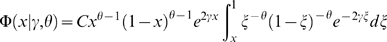
(4)where *C* is chosen so that 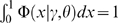 and 0<*θ*<1.

Equations (3) and (4) provide an analytic approximation for the expected dN/dS ratio between sequences sampled from a single population, which we denote *ω*_pop_:
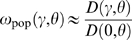
(5)This equation is the single-population analogue of the relationship between selection and dN/dS across long divergent lineages (Equation 2). Note that over long time-scales *ω* depends only on *γ*, whereas within a population *ω*_pop_ depends on both *γ* and *θ*.

### Comparison of dN/dS over Long and Short Time-Scales

**Figure 1:**
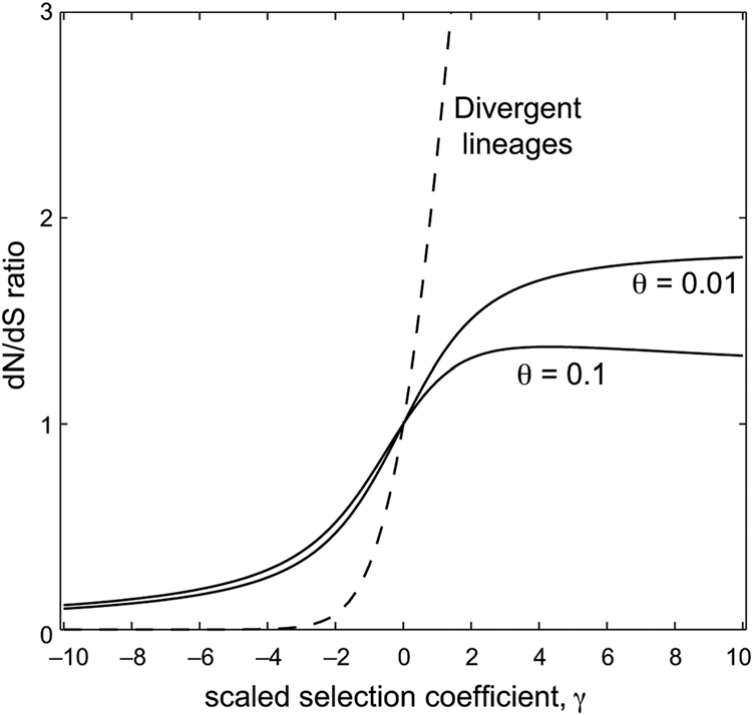
The relationship between the scaled selection coefficient, *γ*, and the expected dN/dS ratio. The dashed line shows the expected dN/dS ratio for samples from divergent lineages, given by Equation (2). The solid lines show the expected dN/dS ratio for within-population samples, given by Equation (5), under two mutation rates.

Across divergent lineages there is a simple monotonic relationship between the selection coefficient, *γ*, and the expected dN/dS ratio, *ω* (Figure 1). A dN/dS ratio less than unity occurs only under negative selection; and a dN/dS ratio greater than unity occurs only under positive selection. Moreover, the dN/dS ratio is very sensitive to the selection coefficient: for *γ* less than −4, the expected dN/dS ratio is near zero (less than 0.01); and the dN/dS ratio climbs very rapidly for *γ* positive.

Within a single population, however, the relationship between selection and dN/dS is markedly different (Figure 1). In the case of negative selection, for example, the expected dN/dS ratio is relatively insensitive to changes in *γ*. Selective constraints that induce a very low dN/dS value when comparing divergent lineages will produce a less extreme dN/dS value when comparing conspecific samples. For example, very strong negative selection (e.g. *γ* = −10) produces an expected dN/dS ratio near zero when comparing divergent lineages, but it produces dN/dS near 0.1 when comparing individuals from a single population. Therefore, the interpretation of an observed dN/dS ratio near 0.1, which is commonly found in practice, depends critically on the time-scale of sequences being compared: within a population such an observation is consistent with strong negative selection, whereas between divergent species such an observation implies weak negative selection.

**Figure 2.**
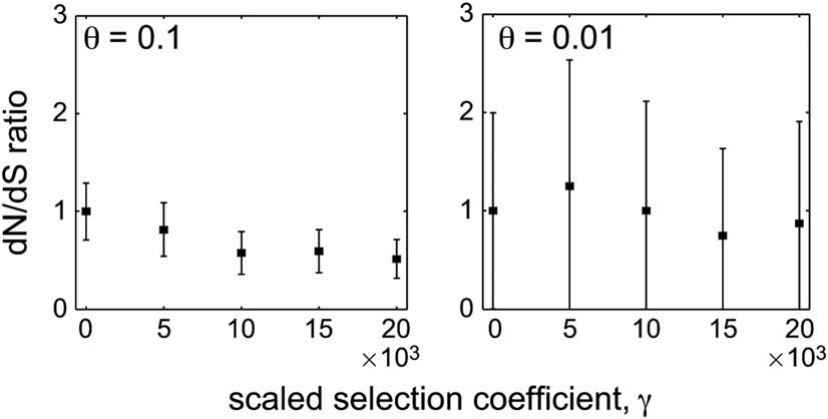
The behavior of the within-population dN/dS ratio for large *γ* in simulated Wright-Fisher populations. Black squares show the mean±two standard errors of the observed dN/dS ratio. Left panel shows results for *θ* = 0.1; right panel shows results for *θ* = 0.01. Simulations were performed at *L* = 10^3^ independent sites.

The difference between short and long time-scales is even more striking in the case of positive selection. Within a population, the dN/dS ratio equals 1 under neutrality (*γ* = 0), as usual. But the dN/dS ratio is not a monotonic function of the selection coefficient: for positive selection of moderate strength the expected dN/dS ratio exceeds one, but as *γ* increases further the dN/dS ratio reaches a maximum value and then starts to descend (Figure 1). In fact, as a standard asymptotic analysis of Equation (5) shows, the expected dN/dS ratio approach zero as *γ* gets very large. This behavior is verified by Figure 2, which shows that dN/dS falls below unity under very strong positive selection. The exact behavior of dN/dS depends upon the mutation rate (Figures 1 and 2), but in all cases the relationship is non-monotonic.

Compared to the case of divergent lineages, the behavior of dN/dS within a population is so radically different that inferences of positive and negative selection based on dN/dS are problematic or, in many cases, impossible. Whereas dN/dS<1 is a faithful indication of negative selection across divergent lineages, the observation of dN/dS<1 within a population is consistent with either weak negative or strong positive selection. The intuition behind this result is straightforward: strong positive selection within a population will produce rapid sweeps at selected sites (but not at neutral sites, which are assumed independent). As a result, two individuals sampled from such a population are likely to contain the same allele at each selected site, producing a dN/dS value less than unity. By contrast, selective sweeps along divergent lineages will tend to produce fixed differences between representative individuals sampled from the two independent populations. Thus, the simple interpretation of dN/dS that applies to divergent lineages does not apply within a population.

### Numerical Simulations

**Figure 3:**
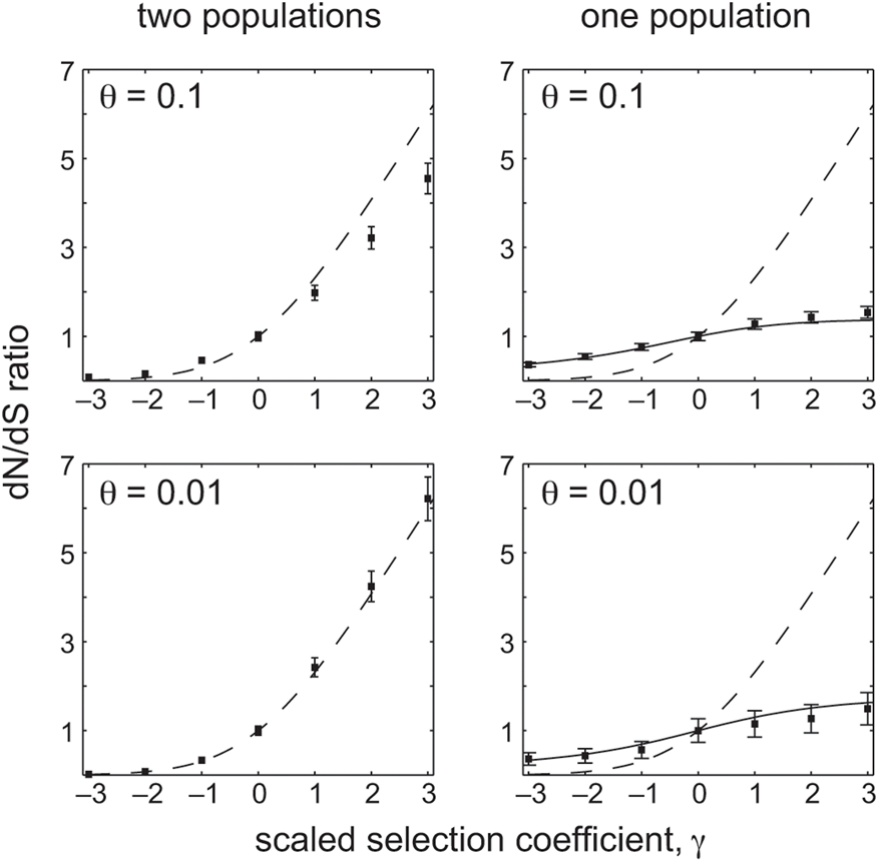
The relationship between the scaled selection coefficient, *γ*, and the dN/dS ratio in simulated Wright-Fisher populations. Black squares show the mean±two standard errors of the observed dN/dS ratio. The predicted dN/dS ratios for divergent lineages are shown in dashed lines (Equation 2); the predicted dN/dS ratios for a single population are shown in solid lines (Equation 5). Left column corresponds to results for two independent populations; right column corresponds to results for a single population. Top panels show results for *θ* = 0.1; bottom panels show results for *θ* = 0.01. The simulations for two populations were performed at *L* = 10^3^ independent sites, and the simulations for a single population were performed at *L* = 10^4^ independent sites.

We performed two sets of Monte Carlo simulations, each based on the Wright-Fisher model with continual selection (i.e. selection *sensu* Yang), for comparison with our analytical results on dN/dS. In the first set of simulations we considered sites that could each assume one of two allelic types, similar to the setup used in our analytical treatment above. We performed a simulation of a single population over a short time-scale, as well as a simulation of two independent populations over a long time-scale (see Methods for details). At the end of each such simulation we sampled a pair of individuals, either from a single population or from each of two independent populations and computed the number of mutations (in the case of single population simulation) or substitutions (in the case of two population simulations) on the lineage separating the two sampled individuals. We compared the observed dN/dS values to their theoretical expectations derived above. Figure 3 summarizes the results of these simulations for two values of the mutation rate and across a range of selection coefficients. In the case of a single population, the observed dN/dS value between sampled individuals agreed very well with our theoretical expectation (Equation 5). In the case of two independent populations, the observed dN/dS value agreed with the expectation derived by Neilsen & Yang (Equation 2). The slight departures between the simulations and Equation (2), visible only at *θ* = 0.1, arise because the theoretical expectations were derived under the assumption that one mutant lineage would fix or go extinct before another mutant lineages is introduced. If we artificially depress the mutation rate to zero whenever two allelic types are segregating in a population we find perfect agreement between theory and simulation, even for *θ* = 0.1 (Figure S1).

The simulation results confirm our theoretical analysis of dN/dS. The relationship between selection and dN/dS is accurately described by Equation (2) when comparing individuals sampled from two divergent lineages. By contrast, when individuals are sampled from a single population, the relationship between selection and dN/dS is radically different and accurately described by Equation (5) —even though the simulation procedure used for a single population is identical to the procedure used in each of the two independent populations.

In the second set of simulations we considered a slightly more realistic situation based on the true genetic code. These simulations employed the same Wright-Fisher model with continual selection, but in this case 64 allelic types are available instead of two. We compared two sampled individuals, each consisting of 10^4^ (single population) or 10^3^ (two populations) independent codon sites, and we estimated dN/dS from the sampled sequences using the PAML computer package, as opposed to using the exact ancestry. Thus, these simulations and dN/dS values provide a close representation of data that are likely to be encountered in practice.

Table 1 summarizes the results of the codon-based simulations. As expected, when comparing sequences from two independent populations the estimated dN/dS value increased monotonically with *s*. Moreover, based on the 95% confidence intervals, dN/dS>1 was rejected in the cases of simulated negative selection (*γ* = −2 or *γ* = −5); and dN/dS<1 was rejected in the cases of simulated positive selection (*γ* = +2 or *γ* = +5). In other words, when comparing divergent lineages the magnitude of dN/dS compared to unity is a faithful indicator of the sign of selection. By contrast, when comparing sequences sampled from a single population, dN/dS did not provide a reliable indicator of the strength or sign of selection, even though the length of the sampled sequences was 10 times larger in the single population simulations than in the two population simulations: for both *γ* = −2 and *γ* = −5 PAML did not reject the possibility that dN/dS>1; and for both *γ* = +2 and *γ* = +5, PAML did not reject the possibility that dN/dS<1. In fact, in one case of simulated positive selection the most likely estimate of dN/dS was less than unity.

**Table 1:** The relationship between the scaled selection coefficient, *γ*, and the dN/dS ratio as estimated by the PAML package from simulated data.

	Two populations	One population
*γ*	*ω*	*ω*_pop_
−5	0.002 (0.000, 0.014)	0.001 (0.000, 2.755)
	0.002 (0.000, 0.014)	0.289 (0.068, 0.813)
−2	0.068 (0.040, 0.106)	1.000 (0.000, 19.300)
	0.105 (0.065, 0.159)	0.608 (0.226, 1.399)
0	0.934 (0.712, 1.237)	0.750 (0.000, 11.020)
	1.066 (0.810, 1.412)	0.967 (0.456, 1.934)
2	4.114 (2.821, 5.451)	0.500 (0.025, 5.621)
	3.245 (1.840, 4.868)	1.472 (0.749, 2.796)
5	4.409 (2.942, 6.172)	2.501 (0.396, 14.330)
	2.823 (1.763, 4.023)	1.680 (0.927, 3.024)

Wright-Fisher simulations based on the full genetic code were performed as described for two independent populations (middle column) and a single population (right column). The table shows the most-likely dN/dS value as estimated by PAML for two sampled sequences, as well as a 95% confidence interval obtained from the *χ* ^2^ distribution. For each value of *γ*, the first line corresponds to simulations with *μ* = 10^−7^, and the second line corresponds to simulations with *μ* = 10^−6^.

The framework used in our second set of simulations is more realistic than the simple two-allele framework used in our theoretical analyses or those of Nielsen & Yang [3]. These simulations demonstrate the generality of our results: when applied to a single population, dN/dS is not particularly sensitive to the strength of selection and it is not a reliable indicator of the sign of selection.

## Discussion

The dN/dS ratio remains one of the most popular and reliable measures of evolutionary pressures on protein-coding regions. Much of its popularity stems from the simple, intuitive interpretation of dN/dS<1 as negative selection, dN/dS = 1 as neutrality, and dN/dS>1 as positive selection. However, this simple interpretation requires that the sequences being compared represent stereotypical samples from divergent populations—an assumption that is also implicit in the methods that estimate dN/dS by maximum likelihood [7]. As we have demonstrated here, the relationship between selection pressure and dN/dS for samples within a population is radically different than the relationship for samples from divergent populations. In particular, within a population dN/dS does not increase monotonically with *γ*, dN/dS is less sensitive to changes in *γ*, and dN/dS<1 can occur under both negative and positive selection.

Recently, Rocha et al. have investigated the relationship between divergence time and dN/dS [44]. Those authors considered an infinite-sites model under negative selection, and they presented an expression for the expected dN/dS ratio in an infinite population. By contrast, we have derived an analytic relationship between the selection pressure and dN/dS at a site under the Wright-Fisher model of a finite population, for both negative and positive selection.

The fact that polymorphisms within a population differ from divergences between species is well understood by population geneticists [23],[45]. However, this important fact is often neglected in many applications of dN/dS to population data. In fact, one recent study explicitly suggests that dN/dS within a population should be used as a surrogate for dN/dS across divergent species [46]. Moreover, the standard infinite-site analysis of neutral and selected segregating polymorphisms (e.g. [23],[47]) would suggest that the ratio 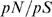 approaches 2 as *γ* gets large, whereas in fact the dN/dS ratio within a population approaches zero for strong positive selection (Equation 5). This discrepancy arises because the infinite-site analysis considers only the mean time that an allele spends in each frequency class while segregrating. By contrast, the single-site analysis (Equation 4) accounts for for the increased amount of time that a site spends in the monomorphic state as *γ* gets large.

Our analysis of selection and dN/dS has assumed independence of sites or, equivalently, free recombination between sites. This assumption is unrealistic in many practical settings. However, the same assumption has been made in prior analytic work on dN/dS [3], and the assumption is expected to be more accurate for small mutation rates, or for weak selection pressures. Outside of this parameter regime, the effects of linkage on dN/dS are difficult to analyze, and they form an important topic for further study.

We have focused our analysis on Yang's particular formulation of selection, which stipulates that all mutations experience the same selection coefficient compared to the resident type [3],[4],[36],[40]. Alternative formulations of selection (e.g. those that assume a constant fitness for each allele) can produce different relationships between *γ* and dN/dS over long time-scales [3]. Our results here, however, do not arise because we have considered a different selective model than Nielsen and Yang [3]; we are studying the same model, but considering samples from a single population instead of divergent populations.

Complications associated with interpreting dN/dS for population samples do not arise in many practical applications of dN/dS—i.e. those involving comparisons among divergent species. However, as sequence data are increasingly available, there is a temptation to apply computer packages such as PAML to intraspecific data—as has been done in many cases already (e.g. [3], [8]–[18]). Published estimates of dN/dS based on samples from a single population are common for microbes and viruses. Inferences about natural selection drawn from such analyses should be interpreted with caution.

Many empirical studies of genes evolving under negative selection have found quizzical results, which our analysis helps to clarify: dN/dS values for such genes are typically closer to 1 when comparing intra-specific samples as opposed to inter-specific samples. This observation holds for bacterial data [11],[12],[14],[16],[18], for viral samples isolated from a single host versus viral samples isolated from different hosts [13], for closely related viral samples versus distantly diverged samples [48], and for conspecific versus interspecific mammalian sequences [49],[50]. A variety of factors have been suggested to explain the elevation of dN/dS within a population under negative selection [49]: balancing selection, variable population sizes, variable mutation rates, relaxed selective constraint within certain lineages [51],[52], statistical artifacts [53], or the prevalence of slightly deleterious mutations [13],[48],[49],[54],[55]. Our analysis clarifies these systematic empirical observations: elevated dN/dS values among conspecifics is expected under a model of continual negative selection, in which all protein-coding mutations experience the same selective constraint at all times (Figure 1). It is important to note that this explanation does not require us to assume a separate class of weakly deleterious mutations [13],[48],[49] or time-varying selective regimes [56].

Our results also have implications for inferences of positive selection based on dN/dS among conspecific samples. Even when samples come from independently evolving populations, the power of the dN/dS statistic to detect positive selection is low when the majority of sites in the protein evolve under purifying selection [28],[57],[58]. Our results indicate that the power of the dN/dS statistic to detect positive selection is further reduced when samples come from a single population (see Table 1). This lack of power has indeed been observed—and, in some cases, interpreted as a lack of selection—in studies of intrapatient HIV evolution [8],[9],[59] and genetic variation in *Salmonella* Typhi [17],[18].

For higher eukaryotes, the distinction between multiple independent populations versus a single population is usually clearcut: samples from different species represent independent populations, whereas conspecific samples should be treated as arising from a single population (unless they are sampled from regions that have been reproductively isolated for more than 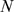 generations). For microbes and viruses, however, the distinction may be more opaque. The central issue is whether or not the sequences being compared represent competing genotypes in the sense of a Wright-Fisher population model. In the case of the human influenza A virus, for example, contemporaneous samples should probably be considered as arising from a single population, because the global population of influenza A strains is known to be well-mixed and genotypes are known to compete for available hosts [60]. When comparing non-contemporaneous samples, however, it is less clear whether the samples should be treated as arising from a single population or independent populations. In some sense, an influenza virus sample from the year 1968 is independent of a sample from year 2000. We might therefore expect that positive selection on influenza's HA locus would produce *ω*>1 when comparing non-contemporaneous samples (independent populations), but *ω*≈1 when comparing nearly contemporaneous samples (single population). This type of pattern has indeed been reported [56], but it was interpreted as a signature of time-varying selection pressures on the HA protein. In fact, this kind of pattern would be expected under continual positive selection, given our analysis of dN/dS over short versus long time-scales.

As the discussion above suggests, it may be difficult to determine the appropriate time-scale associated with a dataset of sampled microbial sequences, particularly for a virus sampled at different timepoints. In fact, there may not be a single time-scale that applies to the entire dataset. In such cases, the relationship between the observed dN/dS ratios and the underlying selection coefficients will be described by some (unknown) mixture of Equation (2) and Equation (5). In such cases our central conclusion still holds: the relationship between selection and dN/dS is not necessarily a simple monotonic function, and it may be impossible to infer the selection pressure from the dN/dS measurement.

## Methods

### Stationary Distribution for a Site under Continual Selection

Here we derive the stationary distribution (4) under Yang's model of continual positive or negative selection. Consider a haploid population of constant size 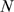, where each individual carries one of the two alleles at the focal site. One allele is the resident and confers fitness 1, the other allele is the mutant and confers fitness 1+*s*. Mutations between the resident and the mutant happen at rate *μ* per generation, and it is assumed that 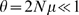. The dynamics of the mutant frequency in the population is described by the classical Wright-Fisher model. Continual selection *sensu* Yang is incorporated in this model by setting the number of mutants to zero as soon as the mutant allele goes to fixation (see main text for details).

Within the standard diffusion approximation, the system is described by the frequency *x* of the mutant allele, which takes values in the interval [0,1]. The probability density *f*(*x*, *t*; *p*) of the mutant frequency to be *x* at time *t* given that it was *p* at the time zero satisfies the forward Kolmogorov equation
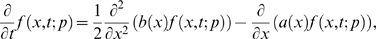
(6)where *a*(*x*) = *γx*(1−*x*)−*θx*/2+*θ*(1−*x*)/2, *b*(*x*) = *x*(1−*x*), and *t* is measured in 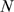 generations. Function *f*(*x*, *t*; *p*) is subject to the following auxilary conditions.
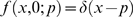
(7)

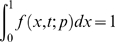
(8)

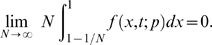
(9)


Equations (7) and (8) are the initial condition and the normalization condition, respectively. The non-standard condition (9) arises in the model of selection *sensu* Yang from the following consideration. In this model, the mutant allele becomes the new resident allele when it fixes in the population. In other words, the population becomes monomorphic for the resident type (the number of mutants is 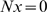) immediately upon the fixation of a mutant (the number of mutants is 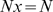). Thus, the probability of finding the population in the state where the mutant is fixed, 
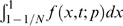
, must tend to zero with increasing 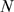. Even though this integral does decays to zero, it must do so at least as fast as 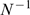 in order for the diffusion approximation to hold. This leads to Equation (9), which is essentially an absorbing boundary condition at *x* = 1. Similar flux conditions have been studied in models of variable selection pressures [43].

It is worth noting that our boundary condition is not the same as a periodic boundary condition. A periodic condition would allow probability flux from state *x* = 1 into state *x* = 0 as well as in the reverse direction–whereas Yang's model of selection should allow only the former direction of flux.

We are interested in the stationary solution Φ(*x*|*γ*,*θ*) of Equation (6) subject to conditions (8), (9). It is easy to show that the general stationary solution of (6) is given by
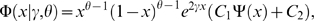
(10)where
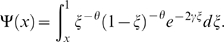
Note that, if we put *C*_1_ = 0, we arrive at the classical zero-flux stationary solution by Wright [26]. However, in Yang's model, the probability flows out of *x* = 1 into *x* = 0, and so we need to satisfy conditions (8) and (9) to determine constants *C*_1_ and *C*_2_. To take the limit in (9), we notice that the following equality is true for any *α*∈[0,1) and any sufficiently smooth function *f*(*x*).
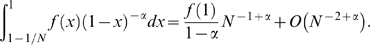
Therefore, putting *f*(*x*) = *x^θ^* ^−1^ *e* ^2*γx*^(*C*_1_Ψ(*x*)+*C*_2_) and *α* = 1−*θ*, we obtain
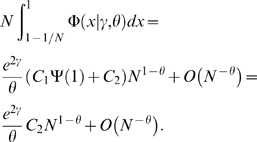
Thus, in order satisfy condition (9), we must require *C*_2_ = 0. This leads to (4) for 0<*θ*<1. A comparison between this stationary distribution and numerical simulations is shown in Figure S2.

### Numerical Simulations

#### Two-Allele Simulations

We performed Wright-Fisher simulations of a population of constant size 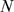 evolving under positive or negative selection *sensu* Yang, in discrete time. In the simulation, each individual carries one of two possible alleles, labeled “0” or “1”. At each generation, one of the alleles, called “the resident”, confers fitness 1, the other allele, called “the mutant”, confers fitness 1+*s* (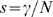 can be negative). However, the labels of the resident and the mutant alleles change over time (see below). During the reproduction round, 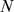 individuals are drawn randomly from the population with replacement, with probabilities proportional to their fitnesses. After choosing which individuals will reproduce, we draw the number of mutations to occur in the replication round from the Poisson distribution with mean 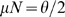, and we randomly assign these mutations to individuals. Since we consider only small mutation rates, typically zero or sometimes one mutation occurs in each generation. A mutation does not create a new allele but rather exchanges the allele label (from “0” to “1” or “1” to “0”) of the individual in which it arises. Once the next generation is formed, we check whether the number of mutant-type alleles has reached 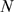, in which case the fitness landscape is reversed: the currently fixed mutant allele becomes the new resident type and it is assigned fitness 1, while the currently absent allele (corresponding to the previous resident) becomes the new mutant type and it is assigned fitness 1+*s*. Thus, the mutant allele always has fitness 1+*s* relative to the resident allele.

This simulation takes the following parameters as input: 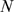, the population size; *s*, selection coefficient; *μ*, mutation rate; *T*, total number of generations; *L*, number of replicate populations or, equivalently, the number of independent sites. We initialized all simulations with a population monomorphic for allele “0”, defined as the initial resident allele. The following parameter values were used for simulations: 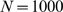 *s* ∈ {−0.003,−0.002,−0.001,0,0.001,0.002,0.003}, *μ* ∈ {5×10^−6^, 5×10^−5^}, These values correspond to *γ* ∈ {−3,−2,−1,0,1,2,3} and *θ* ∈ {0.01,0.1}. We performed simulations of a single population and also simulations of two independent populations, as described below.

*Single population*. We used this type of simulation to test our theoretical predictions for the dN/dS ratio for individuals sampled from a single population. We let a population evolve for 2 *μ* ^−1^ generations in order for it to reach the mutation-selection-drift equilibrium. In the last generation, we sampled two individuals and counted the number of mutations that occurred on the lineage connecting them, *d*(*γ*,*θ*). We compute the mean observed dN/dS value as 
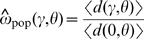
, where 〈*d*(*γ*,*θ*)〉 is the average value of *d*(*γ*,*θ*) over *L* replicate simulations. We compared the observed value 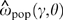 with the theoretically expected value *ω*_pop_(*γ*,*θ*).

*Two divergent populations*. We used this type of simulation to test the predictions for the dN/dS ratio made by Nielsen and Yang [3] (Equation 2) for individuals sampled from two divergent populations. We initialized two populations and let each of them evolve independently for 0.4 *μ* ^−1^ generations, after which we counted the number of substitutions (fixation events) that occurred in each population. The number of substitutions, *s*(*γ*,*θ*), equals the number of mutations that occurred on the lineage connecting the most recent common ancestors of the two final populations. The mean observed dN/dS value is 
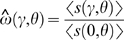
. We compare the observed 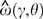 with the theoretical prediction *ω*(*γ*,*θ*).

#### Codon-Based Simulations and Estimation of dN/dS

We also simulated the evolution of a protein coding sequence consisting of *L* independent codon sites, in order to produce data that could be analyzed by the the PAML package [7]. We simulated populations for each site independently. In the final generation of each simulation, two individuals were sampled (either from a single population or from two divergent populations), and the corresponding codons were concatenated. A set of such simulations produces a pair of nucleotide sequences of length 3*L*.

In each simulation at a site, an individual could carry one of the 64 codons. The mutation probability was *μ* per nucleotide per generation. The fitness of an individual was determined by the encoded amino acid: we assumed that only two amino acids, alanine and valine, were allowed at the site; one of them was the resident amino acid and conferred fitness 1, the other was the mutant amino acid and conferred fitness 1+*s*; codons encoding other amino acids or stop codons were assumed lethal (non-reproductive). In all other respects the codon-based simulation was identical to the two-allele simulation. We initiated all simulations with a population monomorphic for codon GTT, which determined the initial resident allele (valine). The following parameter values were used: 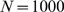 *s* ∈ {−0.005,−0.002,0,0.002,0.005}, *μ* ∈ {10^−7^,10^−6^}. We ran the single population simulations for *L* = 10^4^ sites for *T* = 5×10^5^ generations. We ran the two population simulations for *L* = 10^3^ sites for *T* = 0.25 *μ* ^−1^ generations.

We used the CODEML program from the PAML package to infer the most likely dN/dS ratio for each pair of sequences. We used the likelihood ratio test, based on the *χ* ^2^ distribution, to determine the 95% confidence interval on the estimated dN/dS ratio.

## Supporting Information

Figure S1 The relationship between the selection coefficient, *γ*, and the dN/dS ratio in simulated Wright-Fisher populations for *θ* = 0.1. Mutations are artificially switched off whenever two alleles segregate in the population. Notations as in Figure 2.
(0.3 MB EPS)

Figure S2 Stationary frequency distribution of the mutant allele for the Wright-Fisher model with continual selection. Gray bars show the histrogram obtained from the two-allele simulations with *θ* = 0.1, squares represent the corresponding values expected from Equation (4). Top panel, *γ* = −3, bottom panel, *γ* = 3. Insets show the same data on a different scale.
(0.4 MB EPS)
